# Effects of climatically-modulated changes in solar radiation and wind speed on spring phytoplankton community dynamics in Lake Taihu, China

**DOI:** 10.1371/journal.pone.0205260

**Published:** 2018-10-05

**Authors:** Jianming Deng, Wei Zhang, Boqiang Qin, Yunlin Zhang, Hans W. Paerl, Nico Salmaso

**Affiliations:** 1 Taihu Lake Laboratory Ecosystem Research Station, State Key Laboratory of Lake Science and Environment, Nanjing Institute of Geography and Limnology, Chinese Academy of Sciences, Nanjing, China; 2 Centre for Research on Environmental Ecology and Fish Nutrition (Ministry of Agriculture, China), Key Laboratory of Exploration and Utilization of Aquatic Genetic Resources (Ministry of Education, China), National Demonstration Center for Experimental Fisheries Science Education, Shanghai Ocean University, Shanghai, P. R. China; 3 Institute of Marine Sciences, University of North Carolina at Chapel Hill, Morehead City, North Carolina, United States of America; 4 College of Environment, Hohai University, Nanjing, P.R. China; 5 Research and Innovation Centre, Fondazione Edmund Mach (FEM), San Michele all’Adige, Italy; INRA, FRANCE

## Abstract

Many studies have focused on the interactive effects of temperature increases due to global warming and nutrient enrichment on phytoplankton communities. Recently, non-temperature effects of climate change (e.g., decreases in wind speed and increases in solar radiation) on large lakes have received increasing attention. To evaluate the relative contributions of both temperature and non-temperature effects on phytoplankton communities in a large eutrophic subtropical shallow lake, we analyzed long-term monitoring data from Lake Taihu, China from 1997 to 2016. Results showed that Lake Taihu’s spring phytoplankton biovolume and composition changed dramatically over this time frame, with a change in dominant species. Stepwise multiple linear regression models indicated that spring phytoplankton biovolume was strongly influenced by total phosphorus (TP), light condition, wind speed and total nitrogen (TN) (*r*_adj_^2^ = 0.8, *p* < 0.01). Partial redundancy analysis (pRDA) showed that light condition accounted for the greatest variation of phytoplankton community composition, followed by TP and wind speed, as well as the interactions between TP and wind speed. Our study points to the additional importance of non-temperature effects of climate change on phytoplankton community dynamics in Lake Taihu.

## Introduction

Phytoplankton play essential roles in aquatic food webs and global biogeochemical cycles [1]. Alterations in physical conditions, nutrient inputs, and grazing pressure strongly affect the diversity, structure, and temporal dynamics of phytoplankton communities. Climate change is now recognized a major global driver of changes in phytoplankton community composition and phenological shifts [1, 2].

Most of the phytoplankton papers related to global change emphasize the effect of temperature [3, 4]. The positive selective pressure of increasing temperature for cyanobacteria was well documented [5]. Temperature increase induced by global warming can enhance phytoplankton growth rate directly [6], and warming of surface waters also intensifies vertical stratification, which favors buoyant cyanobacterial bloom taxa [7]. In those lakes with ice-cover in winter, the increase of temperature will reduce the period of ice cover and ice thickness [8]. Jointly, these direct temperature-effects tend to promote cyanobacterial dominance globally [9, 10]. Climate change, however, involves much more than just warming [11]. Other observed climatic variations during last two decades include decline in wind speed [12, 13], changes in rainfall amounts and patterns [14, 15], and increase in solar radiation [16, 17]. These non-temperature factors tend to affect phytoplankton community structure, dominant species and biomass. For instance, in Chesapeake Bay, USA, increases in wind speed enhanced primary production and increased phytoplankton transport to deeper waters in spring by enhancing vertical mixing [18]. Increases in precipitation during winter and spring in Europe have been shown to increase external nutrient loading from catchments [19–21], which enhance primary production. Solar radiation plays a vital role in controlling photosynthesis and has a major role in the melting of lake ice in spring as well [22].

However, most of these studies were carried out in temperate lakes, and there is currently little direct evidence of phytoplankton changes in subtropical lakes attributable to non-temperature related climate impacts; most likely due to a lack of long-term data. In shallow lakes, the sediments are frequently disturbed by wind action, and there is no consistent stratification of temperature and dissolved oxygen in these lakes. Frequent wind-induced resuspension in shallow lakes also markedly increases vertical light attenuation [23]. As a result, light limitation has been reported among these lakes [24]. Wind-induced resuspension will also enhance sediment nutrient release [25]. Despite the recognition that wind stress is one of the most important factors driving mixing and nutrient distribution in the lakes, the response of shallow lakes to long-term wind speed changes has not yet been fully investigated [26]. Changes in rainfall patterns can have multiple effects on phytoplankton community, depending on the characteristics of a particular system [14]. Increased rainfall could lead to a greater nutrient input into the water body through enhanced surface runoff in the watershed [19, 20]. In addition, large storms can play a vital role on destratification of the water column and enhance flushing [27]. Global radiation, which has a major impact on photosynthesis of primary producers, has shown an increasing trend since 1990s, especially during spring in the Lake Taihu basin [16]. It is therefore important to evaluate the relative contribution of both temperature and non-temperature effects of climatic variables on phytoplankton community dynamics in order to understand these combined effects of climate change on different typologies of aquatic systems.

The phytoplankton spring peak has attracted the attention of plankton-related global change research, because it is one of the dominant features in the seasonal growth patterns of phytoplankton [28]. Hence, we evaluated the relative non-temperature effects of climate change on spring phytoplankton community dynamics in shallow, subtropical and, eutrophic Lake Taihu, China. The deteriorating environmental and ecological conditions in Lake Taihu have attracted worldwide attention and are the focus of numerous research efforts [29]. Lake Taihu has some of the earliest and most continuous limnological observations in China. During the past two decades, Lake Taihu’s spring phytoplankton community has varied significantly [30, 31]. High nutrient levels were the main drivers determining dominant spring taxa, while an early warming spring is always followed by earlier and severer bloom in spring, e.g., the cyanobacterial bloom in spring in 2007 [32]. However, to our best knowledge, there are no studies that have contemporaneously examined the relative contribution of both temperature and non-temperature effects on phytoplankton community dynamics in spring in this lake. Accordingly, our working hypothesis is that when nutrient supply is replete, non-temperature effects of climate change will strongly affect phytoplankton community dynamics in addition to direct effects of temperature in spring in Lake Taihu.

## Methods

### Study site and sampling

Lake Taihu is a shallow, eutrophic, subtropical lake (30°55′40′′–31°32′58′′ N and 119°52′32′′–120°36′10′′ E) located in the Changjiang (Yangtze) Delta, one of the most industrialized and densely populated regions in China. The lake has a mean depth of 1.9 m, a maximum depth of 2.6 m, corresponding elevation of 3.0 m a.s.l. [33]. Since the 1980s, rapid economic development in the Lake Taihu basin has resulted in increasing levels of nutrients and other pollutants discharged via tributaries to the lake. As a result, rapid water quality deterioration, accelerating eutrophication, and nuisance algal blooms (*Microcystis* spp.) have occurred regularly since the early 1990’s [34, 35].

Our study mainly focused on the spring season, from March to May. The primary meteorological factors included air temperature, global radiation, wind speed, precipitation, and sunshine duration. Since prior studies have noted that both nitrogen and phosphorus over-enrichment were responsible for blooms in Lake Taihu [35–37], total nitrogen (TN) and total phosphorus (TP) were included in our study. Because the underwater light climate depends on both incident light and the transparency of the water column [38], which was estimated by Secchi depth (SD), the product of these two variables has been used as a simple proxy for the under-water light condition.

**Fig 1:**
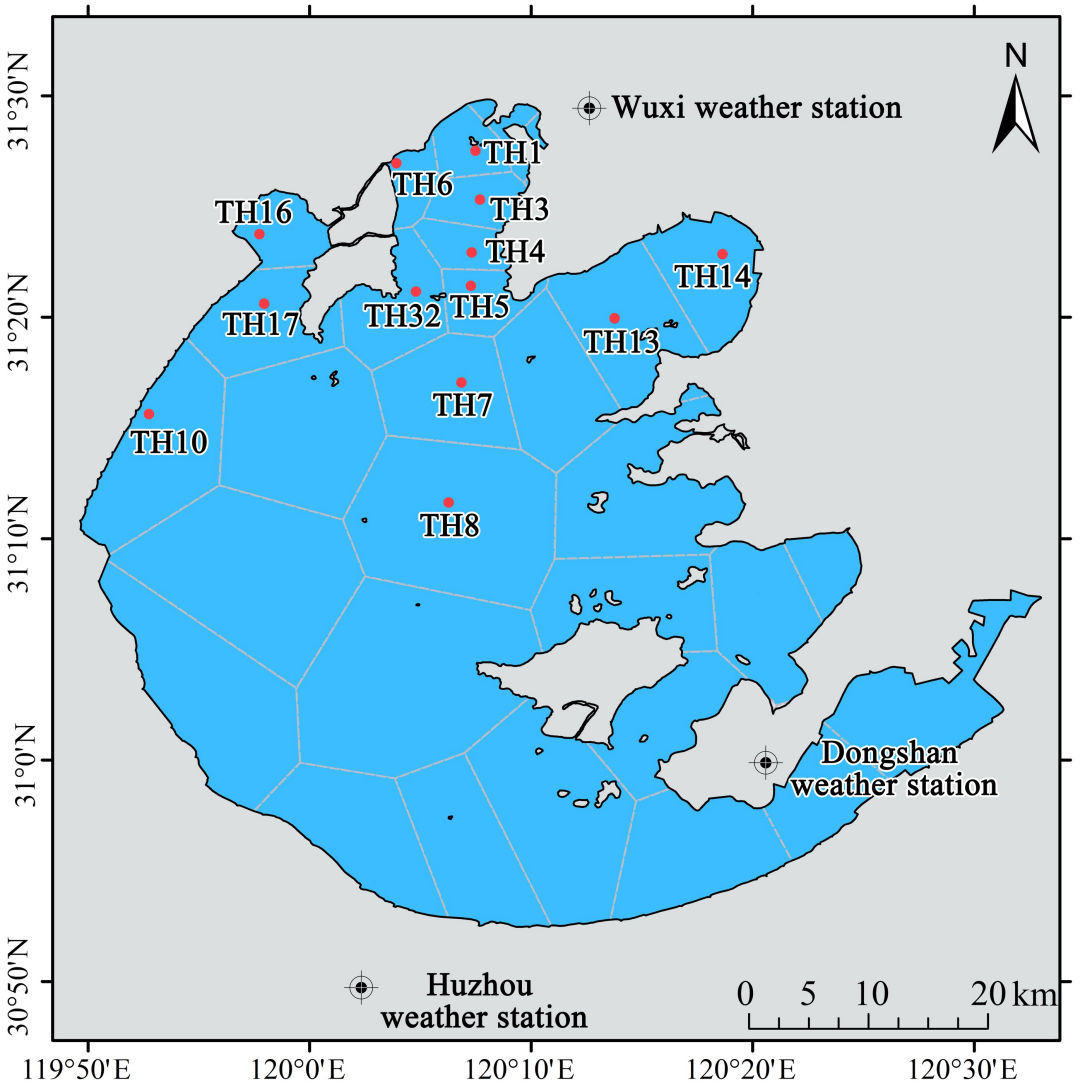
Weather stations and water quality sampling sites in Lake Taihu. Weight factors were calculated based on the relative polygon size to the total area of Lake Taihu. Monthly sampling sites with red dots indicating locations included in the present study. The figure is for illustrative purposes only.

Daily meteorological variables from 1997 to 2016 were calculated as the mean value at three locations, namely Wuxi weather station (station NO. 58354, 31°37′E, 120°21′N), Dongshan weather station (station NO. 58358, 31°04′E, 120°26′N) and Huzhou weather station (station NO. 58450, 30°52′E, 120°03′N), which are located near Lake Taihu (Fig 1). The radiation network in eastern China comprises three stations, Shanghai weather station (station NO. 58367, 31°10′E, 121°26′N), Nanjing weather station (station NO. 58238, 32°00′E, 118°48′N) and Hangzhou weather station (station NO. 58457, 30°14′E, 120°10′N) [39], and mean global radiation values for the stations were calculated. The meteorological data were provided by the China Meteorological Data Service Center (http://data.cma.cn/). The datasets were homogenized to account for station relocations [40]. The daily database included 5796 records was processed to create a monthly database.

Routine monthly monitoring of northern part of Lake Taihu started in 1992, but has been conducted on a more regular monthly basis since 1997. Sampling was generally conducted during the middle of each month. Integrated water samples were collected using a 2-m-long, 10 cm diameter plastic tube at each sampling site. Nutrient concentrations (TN and TP) were analyzed following Chinese standard methods [41]. A standard 30-cm diameter Secchi disk was used to estimate SD.

Phytoplankton samples (1 L) were fixed with Lugol’s iodine solution (2% final concentration) and settled for 48 h. Supernatant liquor was removed slowly by a siphon, and 30 mL was retained for measuring cell density [42]. Cell density was microscopically determined with a 0.1 mL Sedgwick–Rafter counting chamber at magnifications ranging from ×400–512. Phytoplankton species were identified according to Hu et al. [43]. Algal biovolume was calculated from cell numbers and cell size measurements.

In order to avoid the bias in the time-series phytoplankton community data due to different microscopists and taxonomists, Morpho-Functional Groups (MFG) proposed by Salmaso and Padisák [44] and Salmaso et al. [45] were used to classify phytoplankton taxa. The criteria adopted to discriminate the groups include the traits that are valuable for characterizing functional aspects of phytoplankton: motility, the potential capacity to obtain carbon and nutrients by mixotrophy, specific nutrient requirements, size and shape and presence of envelopes. This classification was particularly sensitive and appropriate for monitoring the aquatic systems [46]. The full name list of phytoplankton taxa and MFG is showed in Table 1.

**Table 1:** Morpho-Functional groups identified in our study, and short explanations.

Flagellates	Potential mixotrophs	1a-LargeChry	Large Chrysophytes/Haptophytes
		1b-LargeDino	Large Dinophytes
		1c-LargeEugl	Large Euglenophytes
		2a-SmallChry1	Small Chrysophytes/Haptophytes
		2b-SmallDino	Small Dinophytes
		2c-SmallEugl	Small Euglenophytes
		2d-Crypto	Cryptophytes
	Mostly autotrophs	3a-UnicPhyto	Unicellular Phytomonadina
		3b-ColoPhyto	Colonial Phytomonadina
Without flagella	Cyanobacteria	5a-FilaCyano	Thin filaments (Oscillatoriales)
		5b-LargeVacC	Large Chroococcales with aerotopes
		5c-OtherChroo	Other large colonies, mostly Chroococcales without aerotopes
		5d-SmallChroo	Small colonies, Chroococcales
		5e-Nostocales	Nostocales
	Diatoms	6a1-LColCent	Large colonial Centrics
		6a2-LUniCent	Large unicellular Centrics
		6b1-LColPenn	Large colonial Pennates
		6b2-LUniPenn	Large unicellular Pennates
		7a-SmallCent	Small Centrics
		7b-SmallPenn	Small Pennates
	Others-Unicellular	8a-LargeCoCh	Large unicells-unicellular Conjugatophytes/Chlorophytes
		9a-SmallConj	Small unicells-Conjugatophytes
		9b-SmallChlor	Small unicells-Chlorococcales
	Others-Colonial	10a-FilaChlorp	Filaments-Chlorophytes
		10b-FilaConj	Filaments-Conjugatophytes
		11a-NakeChlor	Chlorococcales-Naked colonies
		11b-GelaChlor	Chlorococcales-Gelatinous colonies

In total, there were 300 samples for water quality, and 297 samples for phytoplankton data in our study (spring phytoplankton data for 2004 were not available). Weighted values of nutrient concentrations and phytoplankton biovolume were used in our study to address spatial heterogeneity (Eq 1). Lake Taihu was divided into 32 sections using the Tyson polygon method [47], and a weight factor was calculated as the area of each polygon relative to the total area of the lake for each site (Fig 1). In the study, sites sampled monthly were taken into account.

$$WC\;=\;\frac{\sum_{i\;=\;1}^{n}{C_{i}\times WF}_{i}}{\sum_{i\;=\;1}^{n}{WF}_{i}}$$ (1)

where *WC* was the weighted nutrient concentrations/phytoplankton biovolume for the northern part of the lake in each month; *n* was the number of total sampling sites in the northern part during each month; *WF*_i_ was the weight factor for site *i*; *Ci* was the measured concentrations/biovolume for site *i*.

### Data analysis

The dominant groups were determined by dominance index calculated by Eq (2) [48]:

$$Y_{i}\;=\;(n_{i}/N)\times f_{i}$$ (2)

where *n*_i_ is the total biovolume of each group in all samples; *N* is the total phytoplankton biovolume in all samples; and *f*_i_ is the occurrence frequency of each group. MFGs with *Y*_i_ ≥ 0.02 were selected as dominant groups.

Two dissimilarity indices were used to describe variation in phytoplankton assemblages in spring, i.e., the complement of the Sørensen coefficient (non-metric coefficient) and the Bray & Curtis index [49]. The non-metric coefficient, which is based on presence/absence data, was used to assess taxonomic persistence, i.e., the degree of continuing presence of groups in the assemblages. The Bray & Curtis index, which is based on biovolume data, was used to quantify assemblage stability, i.e., consistency of MFGs structure. All MFGs were included in the calculations.

To explore the relationships among total phytoplankton biovolume and environmental factors, stepwise multiple linear regressions with both forward and backward selection were used [10] by *step* function in R 3.4.1 [50]. Since water temperature responds rapidly to changes in the air temperature in shallow lakes [51], we estimated the water temperature based on the air temperature (S1 Fig), because there were no constant and continuous long-term daily water temperature records for Lake Taihu before 2000. Co-linearity between environmental variables was identified using variance inflation factors (VIF), and the redundant environmental variables with VIF > 10 were removed before analysis. Akaike Information Criteria (AIC) were used to identify the best model [10]. Relative weights were used to determine the independent variables’ contributions to multiple linear regression models. The relative weights were computed by calculating the squared zero-order correlation between the independent variable and the dependent variable and dividing this number by *r*^2^, the method assumes that independent variables are not correlated [52]. Relationships between biovolume of dominant groups and environmental factors were evaluated by Pearson correlations by using the *cor*.*test* function in R 3.4.1.

The influence of the environmental variables on the phytoplankton groups was analyzed by multivariate analyses. Rare groups found in less than four occasions over the whole study period and redundant environmental variables with VIF > 10 were removed before the analyses. Detrended correspondence analysis (DCA) relating biological and environmental data was computed using the “vegan” package [53] to decide whether linear or unimodal ordination methods should be applied. Prior to performing the multivariate analysis, the biotic data were square-root-transformed to better approximate normality. We then performed redundancy analysis (RDA) using the “vegan” package [53] to determine which of the primary environmental variables were responsible for the variation in the phytoplankton community. Significant explanatory variables (*p* < 0.05) were selected using the conservative forward selection method [54]. This procedure was employed using the function *ordiR2step* in the R package “vegan” [53]. Finally, partial RDA (pRDA) was performed to variation partitioning with the significant variables [53].

GAMs generalized additive models (GAMs) [55] were applied by using the “mgcv” package in R 3.4.1 with time-series data according to Boyce et al. [56].

## Results

### Long-term changes of environmental variables

**Fig 2:**
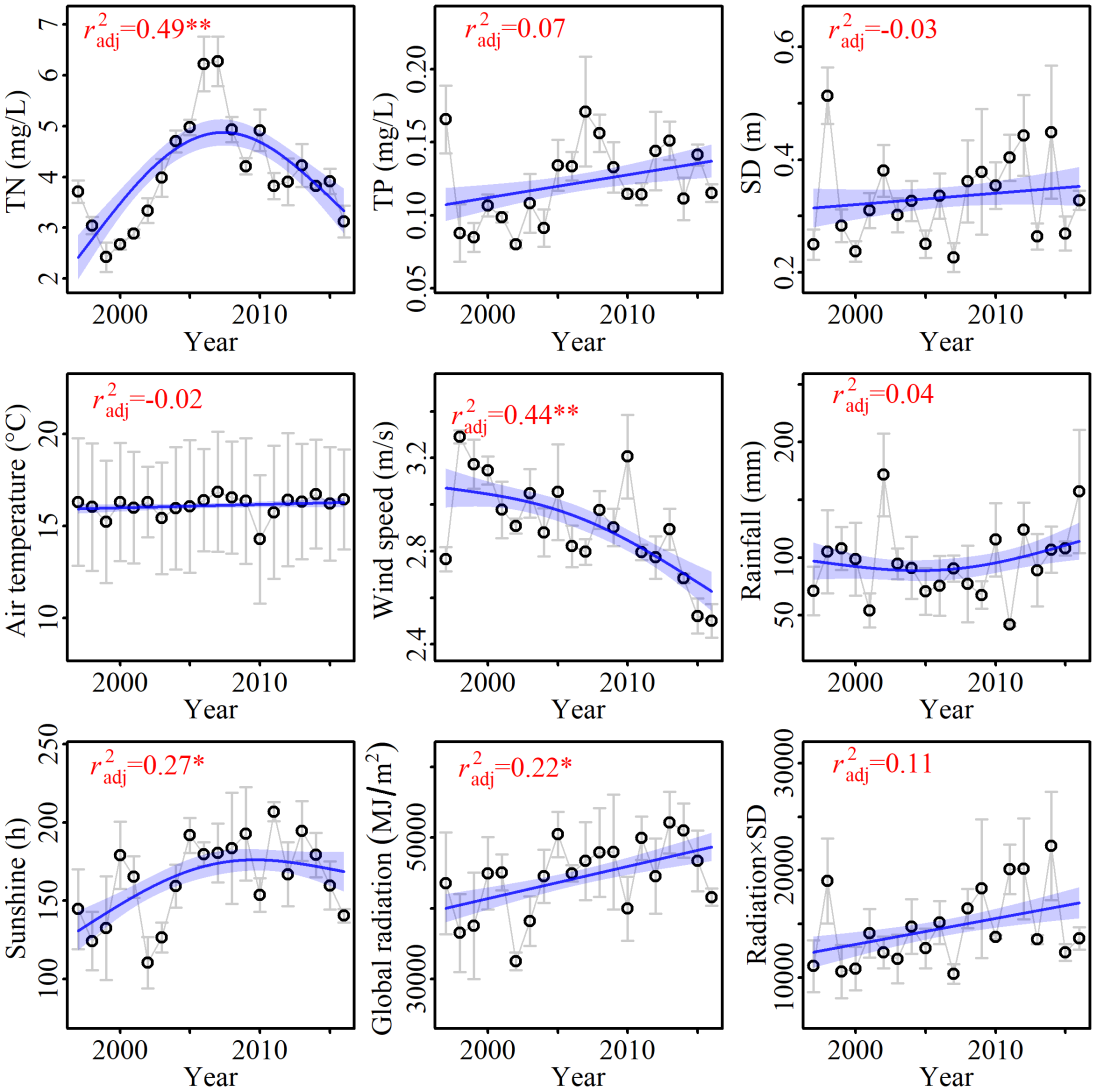
Long-term trends in water quality and meteorological variables Lake Taihu from 1997 to 2016. The trends evaluated by GAMs were shown as blue solid lines. The 95% confidences were shown by blue shade. Adjusted *r*^2^ values were shown. The data were presented as mean ± standard error. * *p* < 0.05; ** *p* < 0.01.

The mean TN concentration was 4.05 ± 1.05 mg/L in spring from 1997 to 2016. TN concentrations increased significantly from 1997 to 2007 and then declined significantly from 2008 to 2016, from 4.9 mg/L to 3.12 mg/L (Fig 2). The TP concentration was 0.12 ± 0.03 mg/L. TP was high in the spring of 1997 (0.16 mg/L) and low in 1998 (0.09 mg/L). TP increased significantly from 1998 to 2007 (it was 0.17 mg/L in 2007) and then declined slightly from 2008 to 2016. Although no distinct tendency was detected for long-term SD variation (*p* > 0.05) using GAMs, a slight increasing trend could be observed from 1999 to 2012.

The mean air temperature in spring was 16.10 ± 0.58°C during the last two decades. The lowest air temperature was 14.28°C, which occurred in 2010. Air temperature showed a non-statistically significant increasing trend from 1997 to 2016. Mean wind speed declined significantly from 3.29 m/s to 2.50 m/s from 1997 to 2016 (*p* < 0.05). The annual amount of rainfall in spring was 95.96 ± 21.46 mm. Rainfall from 1997 to 2009 showed a slight declining trend, but then it increased from 2010 to 2016, though it was not statistically significant.

The amount of sunshine duration in spring was 163.6 ± 26.8 hours from 1997 to 2016. Sunshine hours increased from 1997 to 2011 and then decreased until 2016 (*p* < 0.05). Additionally, global radiation increased from 1997 to 2016 (*p* < 0.05). The variation of radiation × SD during the last two decades showed a slight increasing trend (Fig 2).

### Variation in the spring phytoplankton community in Lake Taihu from 1997 to 2016

**Fig 3:**
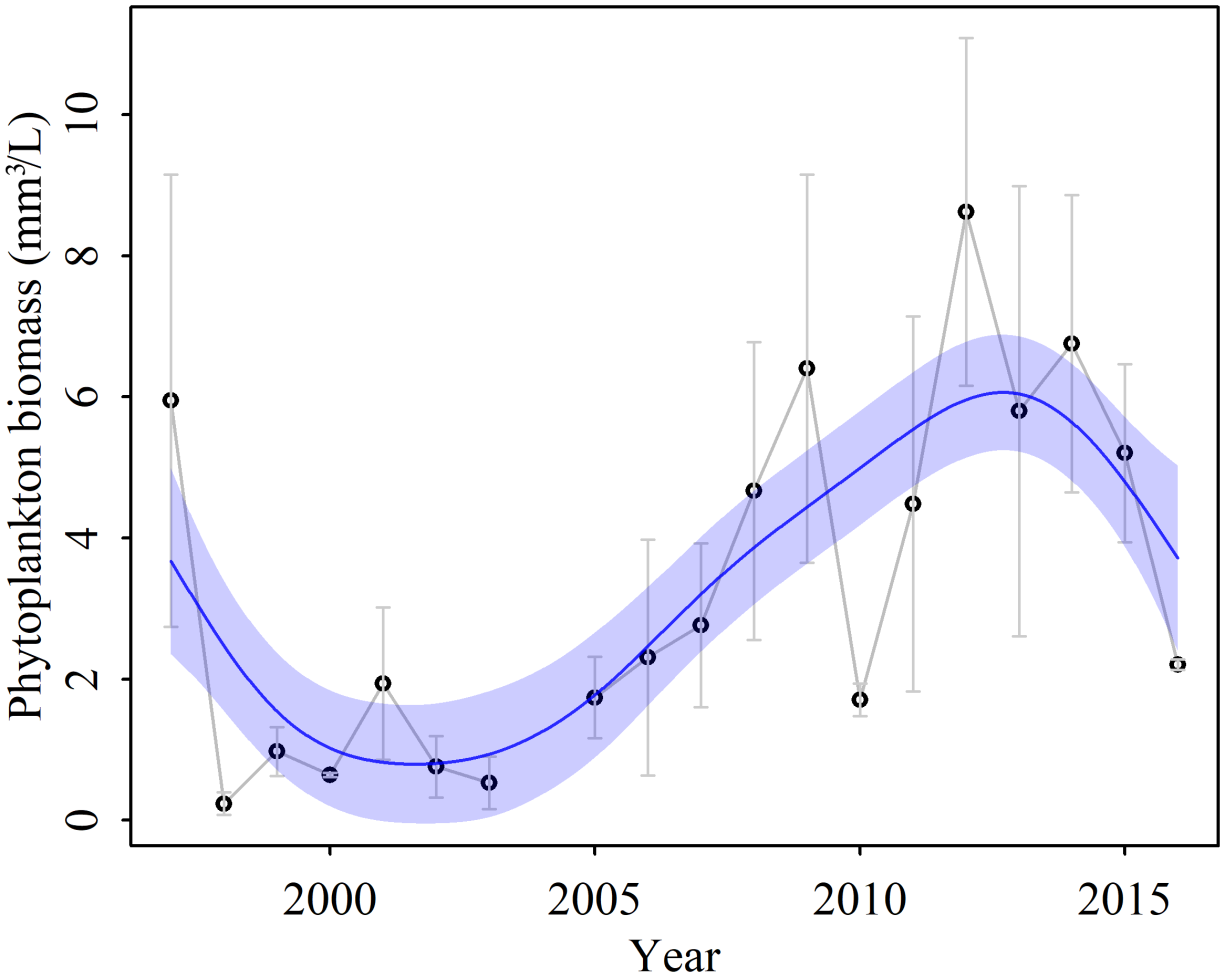
Long-term variation of mean spring phytoplankton biovolume in Lake Taihu from 1997 to 2016. The trends evaluated by GAMs were shown in blue solid line. The 95% confidences were shown by blue shade. The data were presented as mean ± standard error.

The mean spring total biovolume during the past two decades was 3.35 ± 2.53 mm^3^/L. It was high in 1997 (5.95 mm^3^/L) and then dropped to 0.24 mL/m^3^ in 1998. The spring biovolume increased significantly from 1998 to 2012 (slope = 0.42, *r*^2^ = 0.63, *p* < 0.01), and then decreased from 2013 to 2016 (Fig 3).

According to the dominance index, *Y*_i_, 2d (cryptophytes, e.g., *Cryptomonas* spp.), 5b (Large Chroococcales with aerotopes, mainly *Microcystis* spp.), 6a1 (Large colonial centrics, e.g., *Aulacoseira* spp.), 10a (Filamentous chlorophytes, e.g., *Ulothrix* spp.) and 6b1 (Large colonial Pennates, e.g., *Asterionella* spp.) were the five most dominant functional groups (*Y*_i_ > 0.1), and then followed by 6b2 (Large unicellular Pennates, e.g., *Pinnularia* spp.), 11a (Chlorococcales-Naked colonies, e.g., *Scenedesmus* spp., *Pediastrum* spp.), 5e (Nostocales, e.g., *Anabaena* spp., *Aphanizomenon* spp.), 7a (Small Centrics, e.g., *Cyclotella* spp.) and 1c (Large Euglenophytes, e.g., *Euglena* spp.) with *Y*_i_ ranging from 0.02 to 0.05. Biovolume of cyanobacteria (5b, 5e) and diatoms (6a1, 6b1, 7a) increased from 1997 to 2016, while dominance of 2d and 10a decreased during the period (S2 Fig).

According to the non-metric coefficient (top-right in S3 Fig), the spring phytoplankton community from each year shared the same groups, i.e., no new MFGs appeared and no existing MFGs disappeared. However, according to the Bray & Curtis index (bottom-left in S3 Fig), few samples shared the same MFGs composition and structure. The combined information from the Sørensen index and the Bray & Curtis index indicated that the spring phytoplankton community variation in Lake Taihu during the last two decades was caused by the redistribution of biovolume among groups.

### Relationships between community and environmental factors

Among the nine environmental variables, global radiation and SD were excluded in both stepwise regression models and ordination analysis due to collinearity (VIF > 10).

Four variables (light condition, which was estimated as product of global radiation and SD, TP, TN and wind speed) were retained in the best linear regression model: (*r*_adj_^2^ = 0.80, *n* = 19, *F*_*4*,*14*_ = 18.91, *p* < 0.001)

$$\text{Phytoplankton}\;\text{biovolume}\;=\;–1.24+3{.8\times 10}^{-4}\times \text{Light}\;\text{condition}+78\times \text{TP}–0.96\times \text{TN}–2.36\times \text{Wind}\;\text{speed}$$

According to relative weights, TP contributed the most (39.4%) to the overall regression effect (or *r*^2^), followed by light condition (32.8%) and wind speed (21.4%), TN contributed the least to the overall regression effect (6.4%).

There were 10 MFGs with *Y*_i_ > 0.02. Five of them significantly related to light condition (*r* ≥ 0.5, *p* < 0.05, Table 2). Two of the groups were significantly related to sunshine duration (*p* < 0.05), and one group significantly related to wind speed and TP, respectively.

**Table 2:** Pearson correlation coefficients for the biovolume of dominant groups against environmental variables from 1997 to 2016.

Groups	Wind speed	Rainfall	Sunshine	Temperature	TN	TP	Light condition
**2d**	-0.13	-0.25	0.49*	0.27	0.22	0.35	0.53*
**5b**	-0.37	-0.02	0.28	0.36	0.07	0.43	0.09
**6a1**	-0.32	0.05	0.39	0.31	0.12	0.37	0.28
**10a**	-0.19	-0.23	-0.12	0.12	0.10	0.40	-0.25
**6b1**	-0.22	-0.38	0.48*	-0.02	-0.03	0.02	0.59**
**6b2**	-0.41	-0.04	0.38	0.07	-0.03	-0.04	0.68**
**11a**	-0.45	0.22	0.17	0.22	-0.08	0.17	0.50*
**5e**	-0.51*	0.45	0.01	0.09	0.01	0.19	0.21
**7a**	-0.45	-0.09	0.36	0.34	0.12	0.39	0.37
**1c**	-0.28	-0.05	0.45	0.39	0.20	0.47*	0.50*

* *p* < 0.05;

** *p* < 0.01.

First axis length of DCA was 1.6; hence, RDA was chosen in our study [53]. The remaining seven environmental factors explained 56.3% (*F* = 2.02, *p* < 0.05) of the total variance in the phytoplankton community dynamics, while the first two axes collectively explained 45.4% of the variance (axis 1: 34.2%; axis 2: 11.2%).

**Fig 4:**
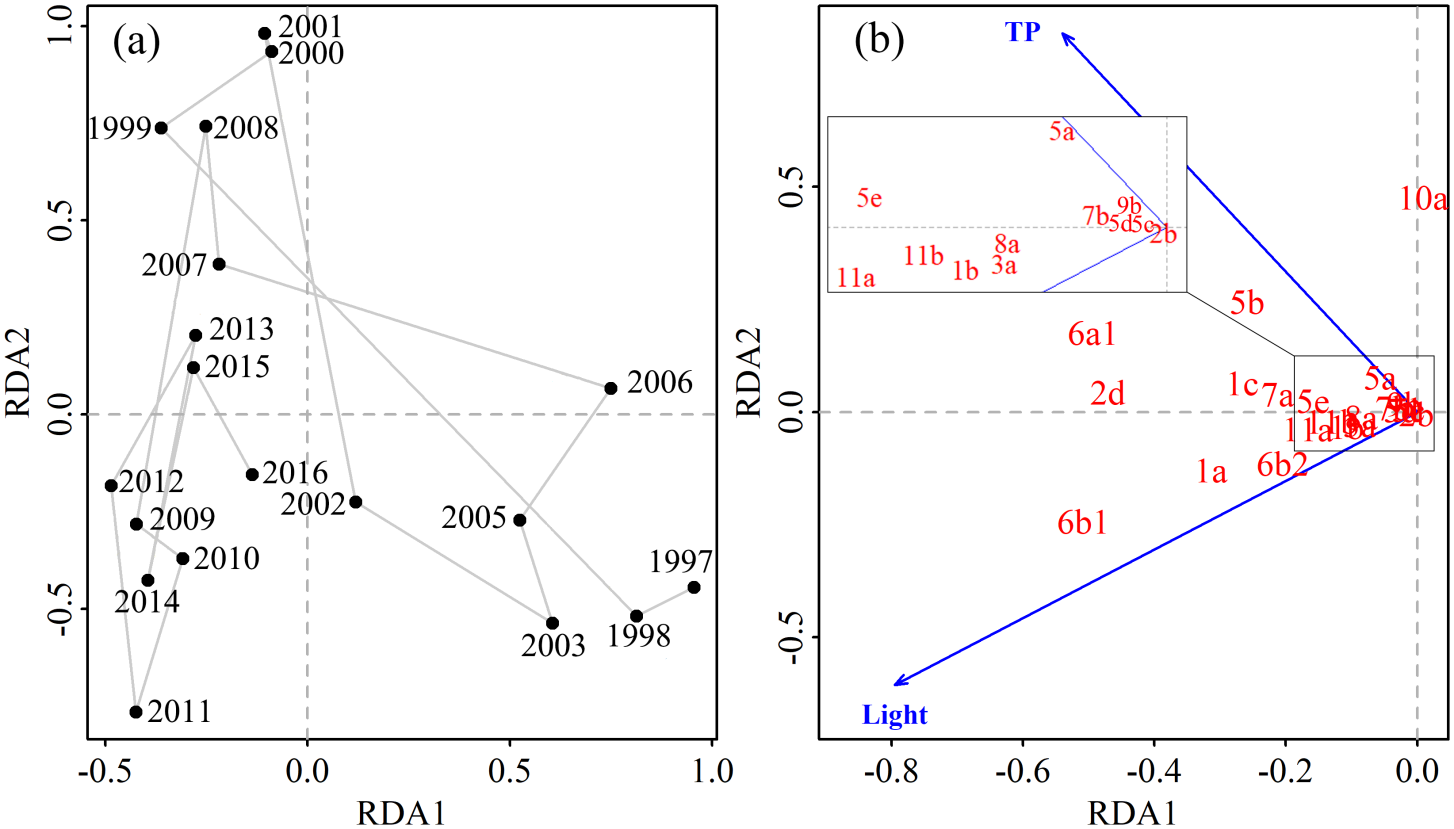
Ordination results. (a) RDA ordination of phytoplankton samples from 1997 to 2016. (b) Environment variables (*p* < 0.05) and MFGs in the first two axes RDA.

The RDA indicated that phytoplankton communities were different among years (Fig 4a). Light condition and TP were the most significant variables related to phytoplankton community variation according to *ordiR2step* procedure (*p* < 0.05; Fig 4b). However, though marginally significant, after inclusion in the RDA analysis, wind emerged as a further important variable (*anova*.*cca*, *p* < 0.1, S4 Fig). The groups most positively and negatively affected by TP (Fig 4b) and wind (S4 Fig), respectively, included 6a1, 5b and 10a. The groups respectively represented by 6b1, 6b2 and 1a were positively affected by light condition. The groups represented by 2d, 1c, 7a, 5c and 11a were positively co-affected by light and TP.

**Fig 5:**
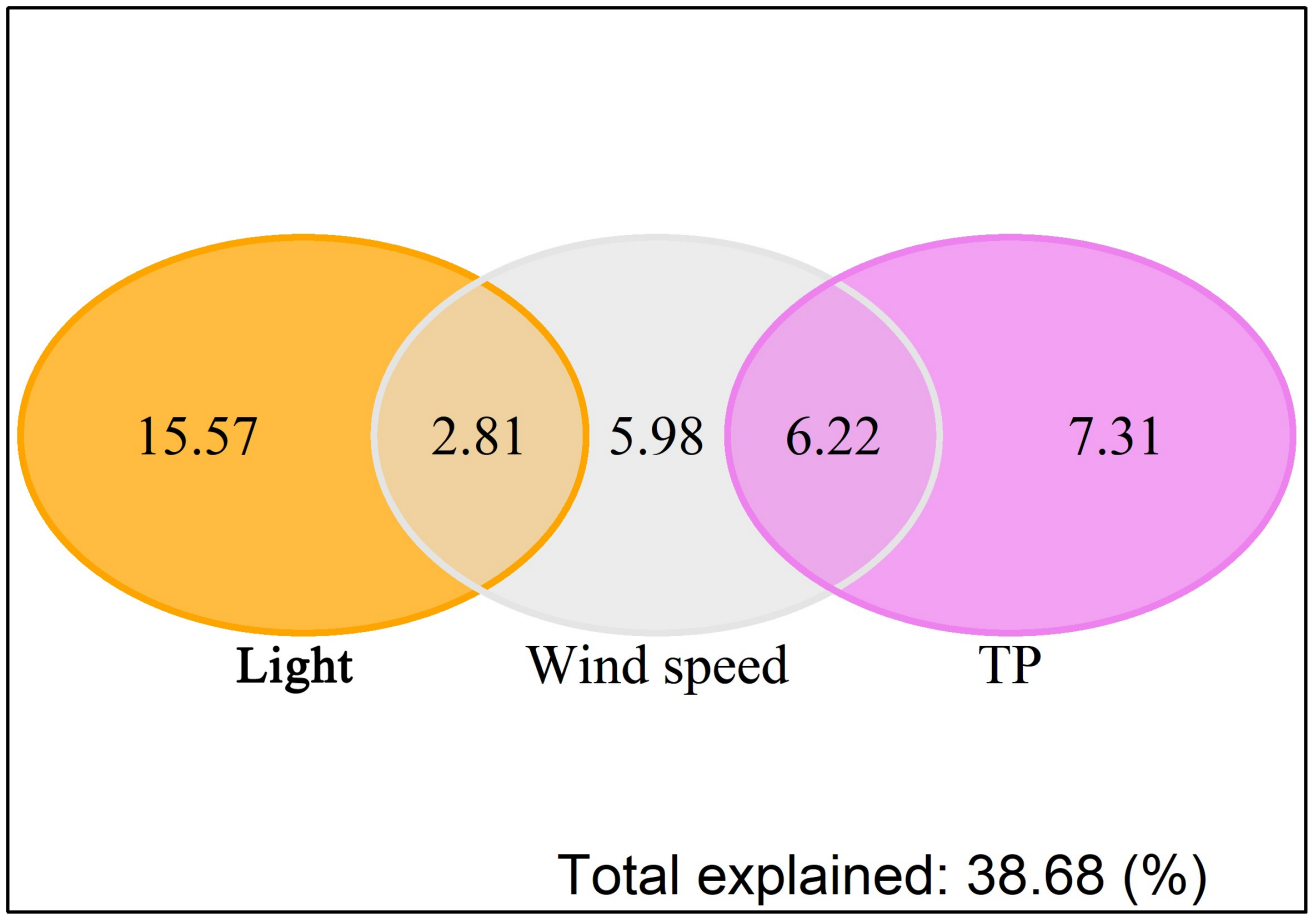
Venn diagram representing the result of the pRDA analyses. Amount of variation in phytoplankton groups biovolume explained by light condition, wind speed, TP and their interactions. Each area is proportional to the share of the inertia explained by the single factor or its interactions with other corresponding factors. Numbers correspond to the percentage of the explained variation associated with each variable type. Negative values are not shown.

The three variables (Light condition, TP and wind speed) explained 36.8% (*F* = 2.9, *p* < 0.01) of the total variance in the phytoplankton community according to pRDA. Light condition alone explained the most total variance in phytoplankton community variation, followed by TP alone, the interaction between wind speed and TP, and wind speed alone (Fig 5).

## Discussion

Lake Taihu’s spring phytoplankton community structure has shifted significantly from 1997 to 2016, while the MFGs present generally remained constant (S3 Fig). Variations in spring phytoplankton assemblages were mainly caused by biovolume redistribution and the exchange of dominant groups. The biovolume of 5b group in spring, which mainly consist of *Microcystis* spp., increased significantly from 1997 to 2016, similar conclusions were drawn by remote sensing study [57]. Since cyanobacterial blooms were of major interest at Lake Taihu, there were few studies focused on the long-term variation of other phytoplankton groups such as diatoms and cryptophytes. Liu et al. [58] and Liu et al. [59] found that diatoms and cryptophytes also dominated occasionally when temperatures were relatively low. In addition, our study indicated increasing trends for diatoms biovolume (6a1, 6b1 and 7a) and decreasing trend for cryptophytes biovolume (2d) in spring after 2006 (S2 Fig).

As demonstrated by RDA, the 6b1, 6b2 and 1a were positively related to light condition (Fig 4b). The frequent mixing events in Lake Taihu could explain biovolume of the settling non-motile diatoms more significantly related to light condition than wind speed in a turbid shallow lake [38]. As indicated by our daily wind speed data from 1997 to 2016, there were 41.4% days with wind speeds > 3 m/s, which could potentially cause sediment resuspension in the northern part [60] and lead to potential light limitation. Light was not a limiting factor for the buoyant cyanobacteria (5b) [61], hence, high nutrients were key factors enhancing cyanobacterial dominance since temperature was relative high in spring in Lake Taihu (mean spring air temperature during last two decades was 16.1 ± 0.6 °C). The motile flagellates 2d can adjust their vertical position to some extent; hence, 2d showed moderate relationships with both TP and light condition. According to our results (S1 Table), the product of global radiation and SD explained the most variance of phytoplankton community dynamics in spring during the last two decades compared to global radiation, sunshine hours, and SD. Most phytoplankton, especially buoyant species (e.g., 5b), are more likely to obtain light near the water surface under turbid conditions in large shallow water bodies like Lake Taihu. According, these species are closely related to the intensity of surface solar radiation. That might be why SD explained fewer variance of phytoplankton community variations in Lake Taihu when compared to global radiation. Another possible reason might be the low frequency of measurements for SD. There was one measurement for each site per month in our study, effects of water transparency on phytoplankton biomass might be underestimated by the low frequency of measurements.

Besides climatic variables, we also included nutrient concentrations in our study. The results showed that, along with wind speed and light condition, TP played an important role on spring phytoplankton community dynamics in Lake Taihu from 1997 to 2016 (Fig 4b), while TN did not show significant relationships with phytoplankton biovolume or community variation. Our results are consistent with *in situ* experiments, which suggest that P was the primary limiting nutrient in spring in Lake Taihu, with no additional effects from N [36].

### Non-temperature effects of climate change in Lake Taihu

Despite efforts to reduce external nutrient loads in Lake Taihu since 2007, blooms have not decreased in magnitude and duration [57]. In fact, our results indicated that cyanobacterial biovolume in spring increased significantly from 1997 to 2016 in the lake (S2 Fig).

The success of cyanobacteria is a result of complex and synergistic environmental factors rather than a single dominant variable [61]. It was further highlighted recently that cyanobacterial blooms are controlled by the synergistic effects of nutrient supplies, light, temperature, water residence time, and biotic interactions [62]. Therefore, temperature, though fundamental, is only one among other factors which mediate phytoplankton community succession. The direct and indirect role of other climatic factors, such as solar radiation and wind, are rarely investigated in much of the recent literature on global climate change [63, 64].

In a few specific cases, such as shallow lakes, the role of light can become important in mediating spring phytoplankton community succession (e.g., [65]). Growth experiments demonstrated that competitive advantage of cyanobacteria can more likely be attributed to their ability to migrate vertically, improve the light supply and prevent sedimentation in strongly stratified waters than temperature [66]. Consistent with these findings, our study also confirmed the climatic effects other than temperature, including light condition and wind speed, can also be important to phytoplankton community succession response in spring in Lake Taihu when nutrient concentrations are relatively high. The importance of wind speed and global radiation on bloom events in Lake Taihu was also reported by remote sensing studies [31].

Compared to nutrients and temperature, the effect of light condition on long-term phytoplankton community structure has received relatively little attention among the studies carried out in Lake Taihu. Remote sensing results indicated that when nutrients were replete, sunshine hours played a vital role in the onset of the blooms in spring [31]. Model results also suggested potential light limitation in Lake Taihu [67]. Light limitation on spring phytoplankton communities were also reported in other water bodies [68, 69]. Modelling studies suggested that phytoplankton growth (e.g., diatoms) in temperate lakes during spring was limited mainly by light [3]. Recently, the updated Plankton Ecology Group (PEG) model [63] emphasized the contribution of light condition to spring blooms in temperate lakes, because phytoplankton growth is more sensitive to light than other factors, e.g., temperature [70]. Light condition not only affects phytoplankton biomass or community directly [71], but also nutrient cycling indirectly in shallow lakes by promoting growth of algae at the sediment and water interface [72], which will affect nutrient fluxes and thus influence the phytoplankton community dynamics.

The effects of wind speed were another climatological factor which has received less attention in comparison to nutrients, and can strongly influence spring phytoplankton biomass (relative weights results) and community structure (Fig 5) in Lake Taihu during the 1997 to 2016 period. The integrated influence of wind speed could be distinguished as having three distinct impacts: (a) direct disturbance impact on phytoplankton cells growths or species composition, due to light fluctuations in the water column [73] and nutrient uptake ability among species [74]; (b) direct transportation impact by both gentle wind-induced surface drift and wave-generated Stokes drift, e.g., Wu et al. [75]; (c) indirect nutrient impact by sediment release. In contrast to general idea that nutrient concentrations positively related to wind disturbance [76] according to short time scale wind events, e.g, [77], our results indicated a negative relationship between annual mean wind speed and annual mean TP in water column (S4 Fig). The explanation could be that wind speed decline in Lake Taihu all year round over the past two decades (Fig 2) would enhance vertical stability of the water column and then enhance phosphorus release from sediment due to low oxygen concentrations in bottom water under stable conditions [78, 79].

## Conclusions

Using long-term monitoring data for Lake Taihu, we reported a shift in the spring phytoplankton community from 1997 to 2016, which was exemplified by a shift in dominant MFGs. The major driver was light condition (e.g., solar radiation and water column transparency), followed by nutrient concentrations and wind speed. Here, we demonstrated that when nutrient supply was replete, non-temperature effects (i.e., light and wind speed) of climate change can significantly impact phytoplankton community biovolume and structure in this large, shallow eutrophic lake.

## Supporting information

S1 Fig Relationship between surface water temperature and air temperature in Lake Taihu. (TIF)

S2 Fig Long-term biomass trends of dominant groups. The trends evaluated by GAMs were shown as blue solid lines. The 95% confidences were shown by blue shade.
(TIF)

S3 Fig Spring phytoplankton assemblage comparison among different years in Lake Taihu. The lower left corner shows the Bray & Curtis index (quantitative dissimilarity coefficient), and the top right corner shows the non-metric coefficients (Sørensen index) computed using mean spring MFGs biomass. Similarity/dissimilarity are shown with colors: green = Sørensen index > 0.7 or Bray & Curtis index < 0.3; blue = Sørensen index > 0.6 or Bray & Curtis index < 0.4; orange = Sørensen index > 0.5 or Bray & Curtis index < 0.5; and red = Sørensen index < 0.5 or Bray & Curtis index > 0.5.
(TIF)

S4 Fig Ordination results with wind speed. (a) RDA ordination of phytoplankton samples from 1997 to 2016. (b) Environment variables (*p* < 0.1) and MFGs in the first two axes RDA.
(TIF)

S1 Table Variation in phytoplankton groups biomass explained by sunshine hours, Secchi disc depth (SD), global radiation and the product of global radiation and SD. (PDF)
