# Supplementary figures and images for: Effects of climatically-modulated changes in solar radiation and wind speed on spring phytoplankton community dynamics in Lake Taihu, China
Source: PLoS One. 2018 Oct 5;13(10):e0205260. doi: 10.1371/journal.pone.0205260 (PMC6173452; doi:10.1371/journal.pone.0205260)

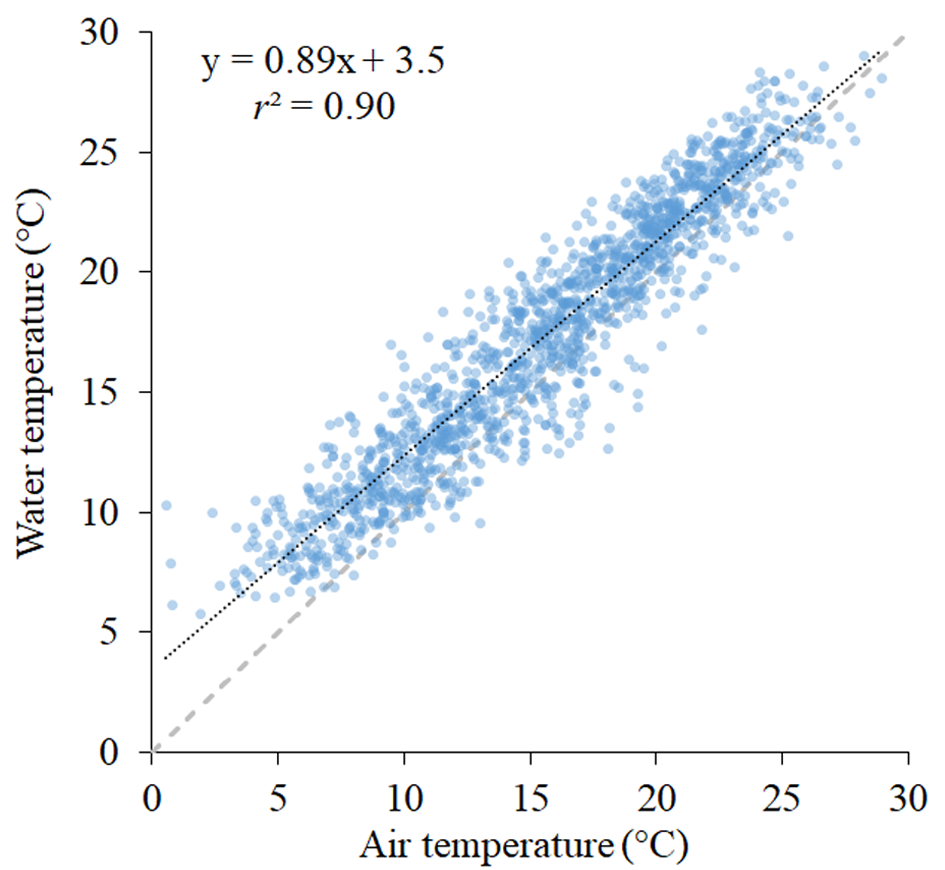

Supplement: S1 Fig — (TIF) [file pone.0205260.s001.tif]

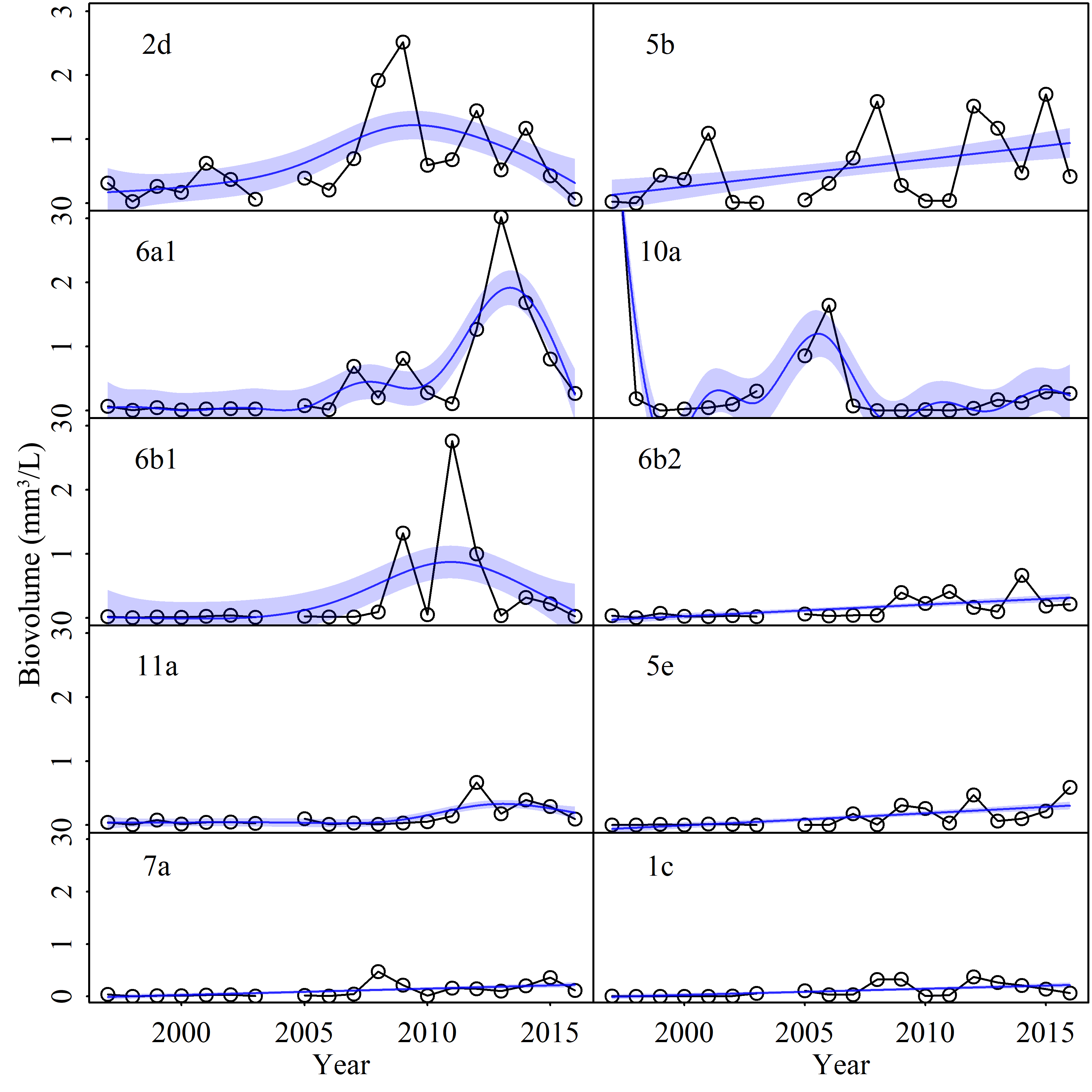

Supplement: S2 Fig — The trends evaluated by GAMs were shown as blue solid lines. The 95% confidences were shown by blue shade. (TIF) [file pone.0205260.s002.tif]

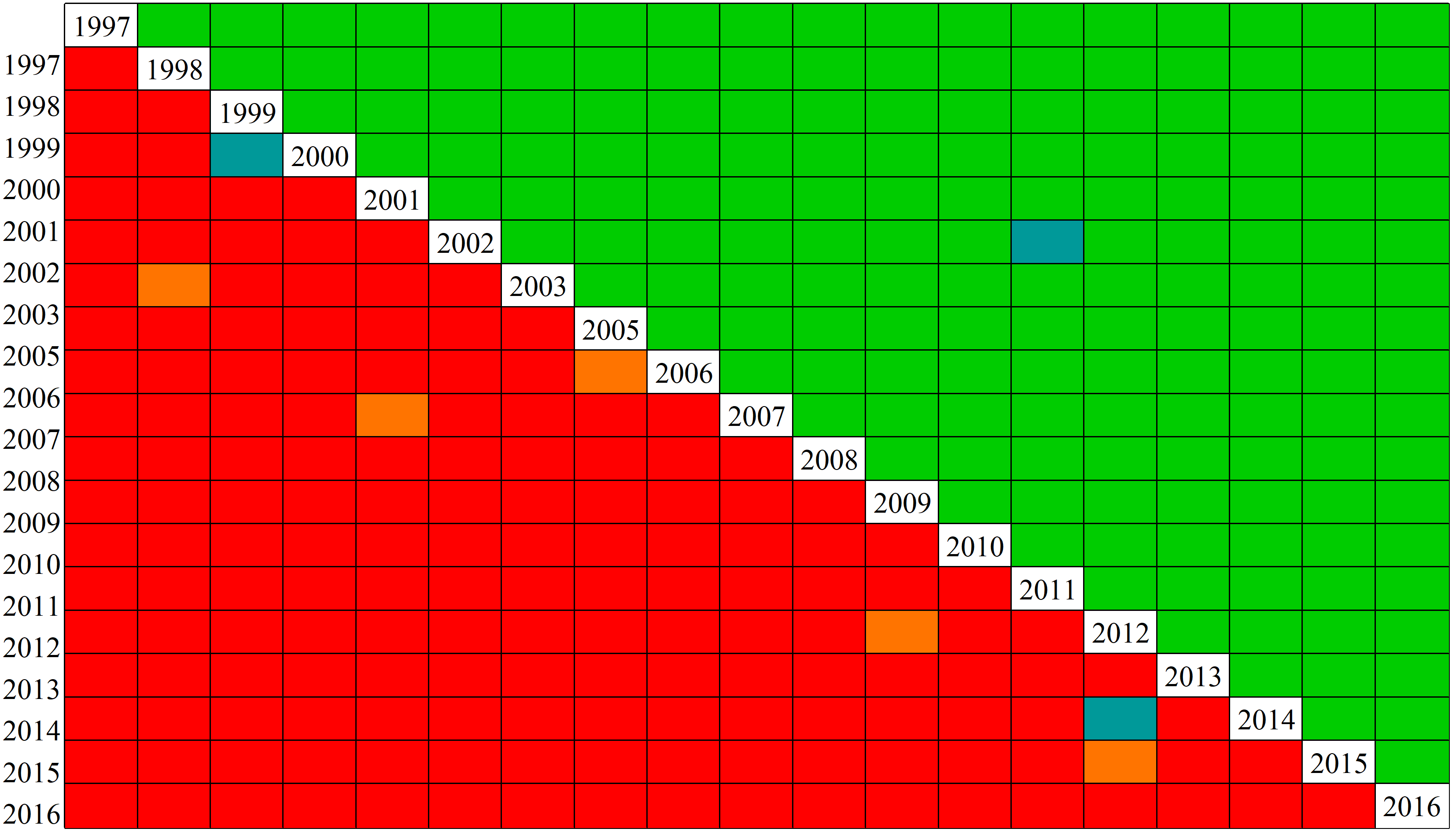

Supplement: S3 Fig — The lower left corner shows the Bray & Curtis index (quantitative dissimilarity coefficient), and the top right corner shows the non-metric coefficients (Sørensen index) computed using mean spring MFGs biomass. Similarity/dissimilarity are shown with colors: green = Sørensen index > 0.7 or Bray & Curtis index < 0.3; blue = Sørensen index > 0.6 or Bray & Curtis index < 0.4; orange = Sørensen index > 0.5 or Bray & Curtis index < 0.5; and red = Sørensen index < 0.5 or Bray & Curtis index > 0.5. (TIF) [file pone.0205260.s003.tif]

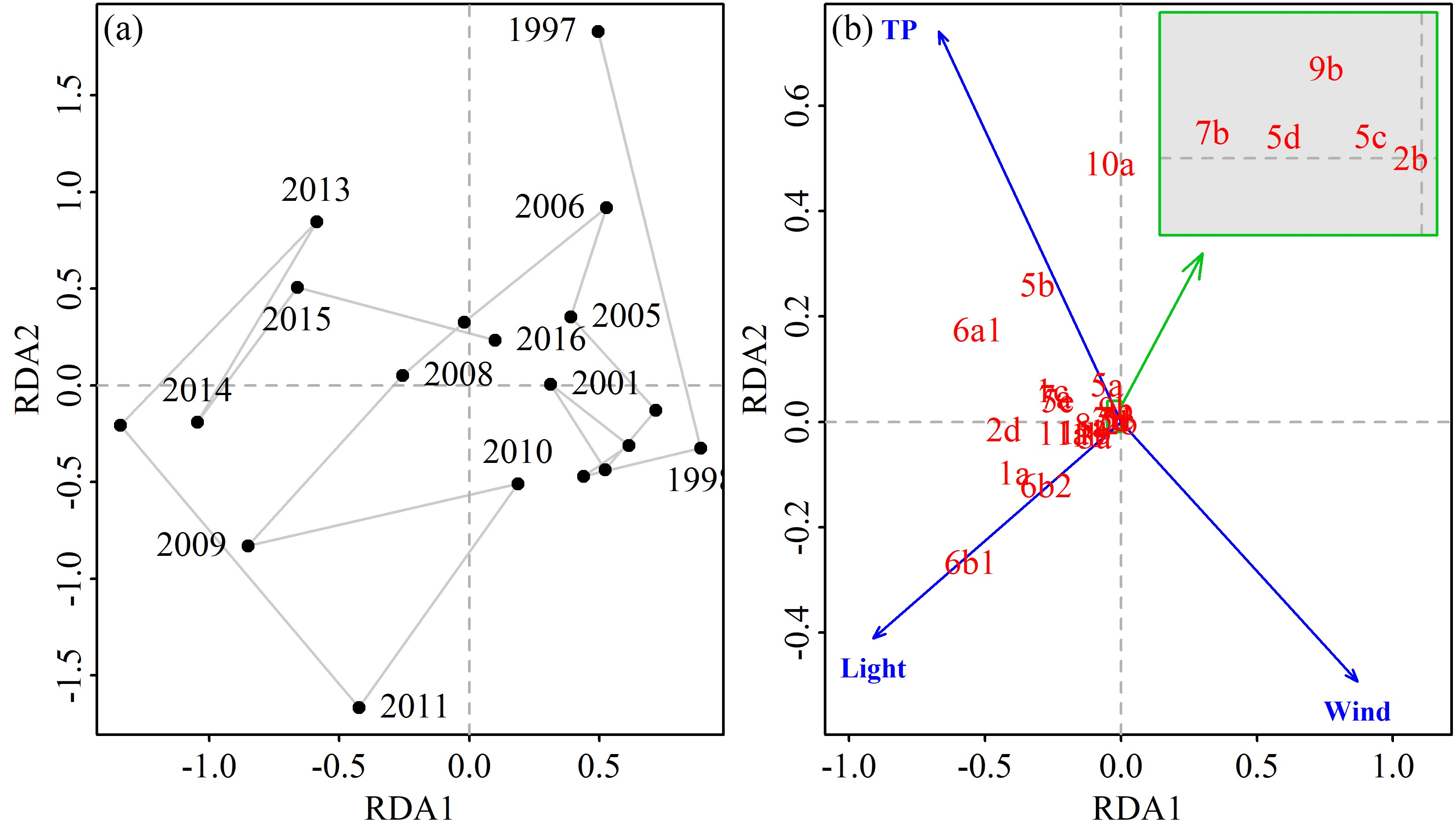

Supplement: S4 Fig — (a) RDA ordination of phytoplankton samples from 1997 to 2016. (b) Environment variables (p < 0.1) and MFGs in the first two axes RDA. (TIF) [file pone.0205260.s004.tif]
