# Supplementary material for: Effects of climatically-modulated changes in solar radiation and wind speed on spring phytoplankton community dynamics in Lake Taihu, China
Source: PLoS One. 2018 Oct 5;13(10):e0205260. doi: 10.1371/journal.pone.0205260 (PMC6173452; doi:10.1371/journal.pone.0205260)
Supplement: S1 Table — (PDF) [file pone.0205260.s005.pdf]

S1 Table

	Sunshine hours	SD	Global radiation	Global radiation×SD
Variance explained (%)	13.8	7.4	16.4	17.3
p	<0.001	<0.05	<0.001	<0.001
